# Associations between food insecurity and psychotropic medication use among women living with HIV in the United States

**DOI:** 10.1017/S2045796020000232

**Published:** 2020-04-06

**Authors:** Henry J. Whittle, William R. Wolfe, Lila A. Sheira, Edward A. Frongillo, Kartika Palar, Daniel Merenstein, Tracey E. Wilson, Adebola Adedimeji, Mardge H. Cohen, Eryka L. Wentz, Phyllis C. Tien, Sheri D. Weiser

**Affiliations:** 1 Centre for Psychiatry, Wolfson Institute of Preventive Medicine, Barts and the London School of Medicine and Dentistry, Queen Mary University of London, Charterhouse Square, London EC1M 6BQ, UK; 2 Department of Psychiatry, University of California, San Francisco (UCSF), San Francisco, CA, USA; 3 Division of HIV, ID and Global Medicine, Department of Medicine, UCSF, San Francisco, CA, USA; 4 Department of Health Promotion, Education, and Behavior, University of South Carolina, Columbia, SC, USA; 5 Department of Family Medicine, Georgetown University Medical Center, Washington, DC, USA; 6 Department of Community Health Sciences, State University of New York Downstate Medical Center, School of Public Health, Brooklyn, NY, USA; 7 Department of Epidemiology and Population Health, Albert Einstein College of Medicine, Bronx, NY, USA; 8 Department of Medicine, Stroger Hospital, Chicago, IL, USA; 9 Department of Epidemiology, Bloomberg School of Public Health, Johns Hopkins University, Baltimore, MD, USA; 10 Department of Medicine, UCSF and Medical Service, Department of Veteran Affairs Medical Center, San Francisco, CA, USA; 11 Center for AIDS Prevention Studies, UCSF, San Francisco, CA, USA

**Keywords:** AIDS, psychiatric services, psychotropic drugs, social and political issues, women

## Abstract

**Aims:**

Psychotropic prescription rates continue to increase in the United States (USA). Few studies have investigated whether social-structural factors may play a role in psychotropic medication use independent of mental illness. Food insecurity is prevalent among people living with HIV in the USA and has been associated with poor mental health. We investigated whether food insecurity was associated with psychotropic medication use independent of the symptoms of depression and anxiety among women living with HIV in the USA.

**Methods:**

We used cross-sectional data from the Women's Interagency HIV Study (WIHS), a nationwide cohort study. Food security (FS) was the primary explanatory variable, measured using the Household Food Security Survey Module. First, we used multivariable linear regressions to test whether FS was associated with symptoms of depression (Center for Epidemiologic Studies Depression [CESD] score), generalised anxiety disorder (GAD-7 score) and mental health-related quality of life (MOS-HIV Mental Health Summary score; MHS). Next, we examined associations of FS with the use of any psychotropic medications, including antidepressants, sedatives and antipsychotics, using multivariable logistic regressions adjusting for age, race/ethnicity, income, education and alcohol and substance use. In separate models, we additionally adjusted for symptoms of depression (CESD score) and anxiety (GAD-7 score).

**Results:**

Of the 905 women in the sample, two-thirds were African-American. Lower FS (i.e. worse food insecurity) was associated with greater symptoms of depression and anxiety in a dose–response relationship. For the psychotropic medication outcomes, marginal and low FS were associated with 2.06 (*p* < 0.001; 95% confidence interval [CI] = 1.36–3.13) and 1.99 (*p* < 0.01; 95% CI = 1.26–3.15) times higher odds of any psychotropic medication use, respectively, before adjusting for depression and anxiety. The association of very low FS with any psychotropic medication use was not statistically significant. A similar pattern was found for antidepressant and sedative use. After additionally adjusting for CESD and GAD-7 scores, marginal FS remained associated with 1.93 (*p* < 0.05; 95% CI = 1.16–3.19) times higher odds of any psychotropic medication use. Very low FS, conversely, was significantly associated with lower odds of antidepressant use (adjusted odds ratio = 0.42; *p* < 0.05; 95% CI = 0.19–0.96).

**Conclusions:**

Marginal FS was associated with higher odds of using psychotropic medications independent of depression and anxiety, while very low FS was associated with lower odds. These complex findings may indicate that people experiencing very low FS face barriers to accessing mental health services, while those experiencing marginal FS who do access services are more likely to be prescribed psychotropic medications for distress arising from social and structural factors.

## Introduction

People living with HIV (PLHIV) experience high rates of mental illness, including elevated rates of depression and anxiety (Weiser *et al*., 2004). In the United States (USA), poverty and social deprivation are concentrated among PLHIV (Pellowski *et al*., 2013), and may contribute to poor mental health. An important challenge that low-income PLHIV in the USA frequently face is food insecurity (Palar *et al*., 2016; Spinelli *et al*., 2017), which includes food insufficiency and hunger, poor quality diets, persistent uncertainty around access to food and having to engage in personally or socially unacceptable food procurement (Jones *et al*., 2013). Food insecurity has been associated with a range of poor mental health outcomes including depression (Whitaker *et al*., 2006; Palar *et al*., 2015; Tuthill *et al*., 2019), anxiety (Whitaker *et al*., 2006; Whittle *et al*., 2019*b*), symptoms of post-traumatic stress disorder (Golin *et al*., 2016; Whittle *et al*., 2019*b*), substance use (Whittle *et al*., 2019*a*) and suicidality (Alaimo *et al*., 2002; Davison *et al*., 2015). While people who experience mental illness likely face more barriers to accessing healthy food, evidence from longitudinal and qualitative studies indicates that food insecurity contributes to symptoms of common mental illness (Hamelin *et al*., 2002; Palar *et al*., 2015; Whittle *et al*., 2016; Tuthill *et al*., 2019; Whittle *et al*., 2019*b*). Provision of food support to food-insecure individuals in a manner consistent with the preservation of dignity has been shown to reduce symptoms of depression (Palar *et al*., 2017; Palar *et al*., 2018).

These findings raise questions about how symptoms of common mental illness occurring in the setting of adverse social and structural factors should be addressed. Mental illness and its treatment are often formulated according to a ‘biopsychosocial’ model (Engel, 1977) in which multidimensional influences on mental health are addressed concurrently through psychotropic medications, psychological interventions and services aimed at improving social circumstances. Yet, in practice, psychotropic medications often predominate. In the USA, data have shown significant upward trends over the past two decades for the use of psychotropic medications alone, compared to significant downward trends for the use of psychotherapy and psychotropic medications together or psychotherapy alone (Olfson and Marcus, 2010). One in six US adults is now prescribed a psychotropic medication, rising to one in five among non-Hispanic White adults and one in four among adults aged 60–85 years (Moore and Mattison, 2017).

Pharmaceutical drugs are prominent for several reasons. Psychotropic medications have the most extensive evidence base among mental health interventions, as their effects can be measured through randomised controlled trials more easily than other forms of intervention. In meta-analyses of trials, common classes of psychotropic medications including antidepressants and antipsychotics show modest but significant therapeutic effects for their respective indications (Leucht *et al*., 2013; Cipriani *et al*., 2018). Prescribing drugs is also less labour-intensive than psychological or social interventions, and often more accessible and time-efficient for service users. In the USA specifically, the market-based structure of the healthcare system may contribute to higher rates of psychotropic medications, which have the financial and promotional backing of for-profit pharmaceutical companies (Moncrieff, 2008). Conversely, reimbursement rates for non-pharmacological treatments by Medicare (US government health insurance for low-income individuals) have been falling steadily for many years, driving psychologists and other allied professionals away from low-income service users (American Psychological Association, 2015). Furthermore, psychotropic medications adhere to a medical model of intervention that accords with the clinical education of prescribers. The paucity of social science training in clinical curricula leaves clinicians lacking the intellectual tools and frameworks to fully understand how social-structural issues may drive distress (Holmes, 2013; Metzl and Hansen, 2014; Hansen *et al*., 2018). Consequently, social interventions may be placed at lower priority than pharmaceutical drugs by default, principally through unfamiliarity and misunderstanding on the part of clinicians.

The vulnerability of public funding for social support to changes in fiscal policies and political ideologies may also contribute to the primacy of pharmacologic interventions. In the USA, public spending on social safety net institutions has undergone a sustained reduction since the 1980s (Harvey, 2005; Wacquant, 2009; Katz 2013; Schram, 2015). The welfare reforms of 1996 had a particularly detrimental effect on the provision of social support, significantly curtailing access to government income for non-disabled adults, with the most severe restrictions targeting those without dependent children (Hansen *et al*., 2014; Whittle *et al*., 2017). Notably, this development has left federal disability income as one of the last forms of substantial government assistance available to many indigent adults in the USA (Whittle *et al*., 2017). Recent studies have suggested that this shift may be fuelling a ‘medicalisation of poverty’, as diagnoses of chronic illness – and particularly mental illness – play an increasingly important economic role for struggling adults to obtain income security through disability status (Hansen *et al*., 2014; Knight, 2015). In this respect, diagnoses of mental illness, accompanied by treatment with psychotropic medications, can act as an important gateway to a level of income stability otherwise unobtainable for many in the current US context of widespread working poverty under welfare reform (Hansen *et al*., 2014; Knight, 2015). Identifying these social and economic realities does not imply that disabled individuals are malingering, or that clinicians are prescribing for non-clinical reasons, but suggests instead that we consider the impact, at a population level, of structural factors that incentivise the prescription of psychotropic drugs for socially deprived individuals.

These arguments raise the question of whether social adversity might drive higher rates of psychotropic prescriptions, independent of psychiatric symptoms. Few empirical studies have attempted to investigate this possibility. We used data from the Women's Interagency HIV Study (WIHS), an ongoing prospective cohort study at nine sites across the USA, to investigate the associations between food insecurity and psychotropic medication use among a broadly representative population of women living with HIV in the USA. Our previous studies in the WIHS cohort have demonstrated dose–response relationships between food insecurity and poor mental health outcomes, including depression (Tuthill *et al.*, 2019), anxiety, stress, symptoms of post-traumatic stress disorder (Whittle *et al.*, 2019b), substance use (Whittle *et al.*, 2019a) and mental health-related quality of life (Tuthill *et al.*, 2019). Here we used a cross-sectional sub-sample of the WIHS cohort for which data on psychotropic medication use were available to test two successive hypotheses: (1) food insecurity would be associated with psychotropic medication use in a dose–response relationship among women living with HIV, mirroring the dose–response relationships between food insecurity and symptoms of common mental illness found in previous studies; and (2) if we additionally adjusted for symptoms of common mental illness, any positive associations between food insecurity and psychotropic medication use would remain significant.

## Methods

### Study design and population

Our study was a cross-sectional analysis of data from the WIHS, a prospective cohort study of HIV-seropositive women and demographically similar HIV-seronegative women in the USA. Cohort recruitment, demographics and retention are described elsewhere (Barkan *et al*., 1998; Bacon *et al*., 2005; Hessol *et al*., 2009; Adimora *et al*., 2018). WIHS participants undergo structured interviews and physical examinations every 6 months at nine sites across the USA and have blood and other biological samples taken. The WIHS began with baseline recruitment in 1994 and has undergone three recruitment waves since. Beginning in 2009, a standardised and detailed neurocognitive assessment was added to the WIHS Core exams and administered every 2 years.

From April 2013 through March 2016, the Food Insecurity Sub-study collected data every 6 months on food security, nutrition and other key socio-economic variables from all WIHS participants. For the current analysis, women living with HIV who participated in the Food Insecurity Sub-study from April 2013 through March 2015 and also had neurocognitive and psychiatric variables (including psychotropic medication use) during the same time period were included (*n* = 905). Data collection for psychotropic medication use was staggered across four WIHS visits during this period, at five study sites: San Francisco, CA; Chicago, IL; Washington, DC; Bronx, NY and Brooklyn, NY.

### Primary explanatory variable

Food security (FS) was the primary explanatory variable, measured using the Household Food Security Survey Module (HFSSM) (US Department of Agriculture, 2012). The HFSSM is an 18-item survey designed to capture the experience of anxiety around household food supplies, inadequate quality of food and/or reduced food intake. Originally developed from in-depth qualitative and survey data among women in the USA (Radimer *et al*., 1992; Wehler *et al*., 1992), it has since been validated in multiple diverse contexts (Jones *et al*., 2013). Respondents are classified as having high, marginal, low or very low FS. Very low FS corresponds to reduced food intake and hunger, while marginal FS implies persistent anxiety and uncertainty around food. The internal consistency of the HFSSM in this sample was high (Cronbach's *α* = 0.90).

### Outcome variables

The primary outcomes were four categories of prescribed psychotropic medication use (antidepressant use, sedative/hypnotic/tranquiliser/anxiolytic use, antipsychotic use and a pooled outcome of any psychotropic medication use). WIHS participants are asked to bring a list of medications to each visit and are also asked specifically whether they are using any medications ‘for psychological conditions or depression’ and for the name of the medication. Self-reported psychotropic medications were coded as antidepressants (e.g. selective serotonin reuptake inhibitors, serotonin–norepinephrine reuptake inhibitors and tricyclic antidepressants), sedatives/hypnotics/tranquilisers/anxiolytics (hereafter referred to as ‘sedatives’; e.g. benzodiazepines and *Z*-drugs) or antipsychotics (e.g. typical and atypical neuroleptics) as appropriate. Using these data, we constructed a pooled outcome for any psychotropic medication use (i.e. use of an antidepressant, sedative or antipsychotic) and made three separate binary outcomes corresponding to each individual drug class.

Other outcomes included symptoms of depression, generalised anxiety disorder and mental health-related quality of life. Symptoms of depression were measured using the Center for Epidemiologic Studies Depression (CESD) score, a widely used self-report instrument that asks participants how often they experience symptoms of depression including low mood, low self-esteem, poor concentration, sleeping difficulties, poor appetite and others (Eaton *et al*., 2004). Scores range from 0 to 60, with higher scores indicating greater depressive symptoms. CESD score is a core WIHS study measure collected from WIHS participants at each visit. The internal consistency of the CESD in our sample was high (Cronbach's *α* = 0.93).

We measured symptoms of generalised anxiety disorder (GAD) using the Generalised Anxiety Disorder-7 (GAD-7) scale, a 7-item self-report instrument used to screen for and categorise the severity of GAD in primary care (Spitzer *et al*., 2006). Participants were asked how often they experience symptoms of GAD including worry, restlessness, irritability and others, with responses scored from 0 to 21. In the WIHS, collection of GAD-7 data only began in October 2013. GAD-7 data were therefore only available for approximately 75% of the women participating in our study. The internal consistency of the GAD-7 in the sample was high (Cronbach's *α* = 0.92).

Mental health-related quality of life was measured using the Mental Health Summary (MHS) score of the Medical Outcomes Study HIV Health Survey (MOS-HIV) scale (Revicki *et al*., 1998). The MOS-HIV scale, a widely used quality of life measure developed and validated among PLHIV, comprises 35 questions across ten domains, providing a total score out of 100. The MHS is calculated from the total MOS-HIV score by means of a standardised method that transforms the scores of relevant domains into a standardised *t*-score with a mean of 50 and standard deviation of 10. MHS is a continuous variable composed of four sub-scales where lower scores indicate worse mental health-related quality of life (e.g. feeling nervous and depressed) and higher scores indicate better mental health-related quality of life (e.g. feeling calm and peaceful). WIHS participants undergo the MOS-HIV annually (i.e. every two study visits). Since psychotropic medication data were staggered across four study visits (approximately 2 years), MHS data were only available for approximately 50% of the women contributing data to this study. The internal consistency of the MHS (Cronbach's *α*) was 0.80.

### Other variables

Based on theory and the literature, we identified potential variables that may confound the relationship between food insecurity and psychotropic medication use (Allen *et al*., 2014). These were age at visit, race/ethnicity (non-Hispanic White, Hispanic, African-American or other), annual income (⩽$12 000, $12 001–24 000 or ⩾$24 001), education (less than high school education *v*. at least high school education), heavy drinking (>7 *v*. ⩽7 alcoholic drinks/week) and illicit substance use (use of any illicit substances not including cannabis since the last visit *v*. none). All of these variables were measured by self-report.

### Ethics statement

Participants provided written informed consent for participation in the WIHS and received compensation for their participation at each visit. The institutional review board of each study site's institution and the WIHS Executive Committee approved this study.

### Statistical analysis

Data were obtained from WIHS visits at which women both completed the HFSSM and had coded psychotropic medication data available, creating a cross-sectional sample staggered over four study visits (approximately 2 years). Initially, we examined associations of FS with common mental illness to confirm whether the dose–response relationships found in previous studies were reproduced in this sub-sample. To examine associations of FS with CESD score, GAD-7 score and MHS score, we ran multivariable linear regressions.

Next, we tested associations between FS and psychotropic medication use in two successive models. First, we ran multivariable logistic regressions to examine associations of FS with any psychotropic medication use and antidepressant, sedative and antipsychotic use individually, adjusting for race/ethnicity, income, education, heavy drinking and illicit substance use. For any psychotropic medication use, antidepressant use and sedative use, we then ran multivariable logistic regressions also adjusted for CESD score and GAD-7 score (in addition to the other variables listed above). We did not include antipsychotic use as an individual outcome in this fully adjusted model because depression and anxiety are not clinical indications for antipsychotic use, which would render the outcome difficult to interpret (the WIHS does not include any measure of psychosis). The area under the receiver operating characteristic curve, which ranges from 0.5 (chance) to 1.0 (perfect), was used to quantify how well the models explained the outcomes (Swets, 1988). All analyses were completed using Stata version 14 (StataCorp LP, College Station, TX, USA).

## Results

There were 905 women in the sample (Table 1). Approximately two-thirds (67.5%) identified as African-American, while just under half (48.6%) reported an annual income less than $12 000. Over one-third (36.8%) were categorised as food-insecure (i.e. marginal, low or very low FS). In total, one-third (33.0%) were taking psychotropic medication. The most common class was antidepressants (taken by 27.6% of women), followed by sedatives (14.0%) and then antipsychotics (9.3%). In adjusted analyses, compared to high FS, marginal, low and very low FS were significantly associated with increasingly higher CESD and GAD-7 scores and with increasingly lower MHS scores, exhibiting a consistent dose–response relationship across all three outcomes (Table 2).

**Table 1.** Background characteristics of sub-sample (*n* = 905)

FS status	
High	572 (63.2%)
Marginal	139 (15.4%)
Low	111 (12.3%)
Very low	83 (9.2%)
Any psychotropic medication use	
No	606 (67.0%)
Yes	299 (33.0%)
Antidepressant use	
No	655 (72.4%)
Yes	250 (27.6%)
Sedative use	
No	778 (86.0%)
Yes	127 (14.0%)
Antipsychotic use	
No	821 (90.7%)
Yes	84 (9.3%)
Age at visit (mean, s.d.)	50.0 (8.6)
Race	
White	116 (12.8%)
Hispanic	142 (15.7%)
African-American/Black	611 (67.5%)
Other	36 (4.0%)
Annual household income	
⩽$12 000	423 (48.6%)
$12 001–24 000	183 (21.0%)
⩾$24 001	264 (30.3%)
Educational attainment	
Less than high school degree	290 (32.1%)
High school degree or higher	614 (67.9%)
Heavy drinking^a^	
No	797 (88.3%)
Yes	106 (11.7%)
Illicit substance use since last visit^b^	
No	829 (91.8%)
Yes	74 (8.2%)

a Heavy drinking defined as ≥7 drinks/week (1 drink defined as 1 can, bottle, or glass of beer; 1 glass of wine; 1 shot of liquor on its own or in a mixed drink; or any other kind of alcoholic beverage).

b Participants were asked if they used cocaine, crack, heroin, methamphetamine, hallucinogens, club drugs, or any other illicit or recreational drugs not including cannabis since the last visit.

**Table 2.** Multivariable linear regressions to examine the association between FS and symptoms of common mental illness among HIV+ women

	CESD score		GAD-7 score		MHS score	
	*β*	95% CI	*β*	95% CI	*β*	95% CI
FS status (high ref)						
Marginal	4.08***	2.14–6.02	1.35*	0.19–2.51	−8.54***	−13.01 to −4.06
Low	8.14***	6.00–10.28	3.38***	2.13–4.63	−10.19***	−15.27 to −5.12
Very low	9.53***	7.08–11.98	3.47***	1.90–5.03	−11.55***	−16.94 to −6.16
Age at visit	−0.02	−0.10 to 0.06	−0.01	−0.06 to 0.03	−0.12	−0.30 to 0.06
Race (non-Hispanic White ref)						
Hispanic	−0.54	−3.14 to 2.06	0.10	−1.45 to 1.65	2.36	−3.72 to 8.45
African-American/Black	−1.37	−3.49 to 0.74	−1.37*	−2.64 to −0.09	7.91**	2.94–12.89
Other	0.29	−3.57 to 4.15	0.99	−1.37 to 3.35	3.66	−5.27 to 12.58
Annual household income (⩽$12 000 ref)						
$12 001–24 000	0.05	−1.73 to 1.83	0.42	−0.63 to 1.46	1.89	−2.36 to 6.14
⩾$24 001	−3.15***	−4.83 to −1.47	−1.32*	−2.34 to −0.31	6.67**	2.67–10.66
Educational attainment						
High school degree or higher	−2.41**	−3.92 to −0.91	−0.31	−1.20 to 0.58	4.55*	0.97–8.14
Heavy drinking^a^						
Yes	0.65	−1.48 to 2.77	0.39	−0.89 to 1.67	2.69	−2.23 to 7.60
Illicit substance use since last visit^b^						
Yes	5.79***	3.24–8.34	2.18**	0.65–3.70	−11.26***	−17.47 to −5.06
Observations	862		659		464	
*R*^2^	0.20		0.13		0.19	

^∗∗∗^*p* < 0.001, ^∗∗^*p* < 0.01, ^∗^*p* < 0.05.

a Heavy drinking defined as ≥7 drinks/week (1 drink defined as 1 can, bottle, or glass of beer; 1 glass of wine; 1 shot of liquor on its own or in a mixed drink; or any other kind of alcoholic beverage).

b Participants were asked if they used cocaine, crack, heroin, methamphetamine, hallucinogens, club drugs, or any other illicit or recreational drugs not including cannabis since the last visit.

Of the other variables studied, self-identifying as African-American/Black, having an income ⩾$24 001, and having at least a high school education were all variously associated with better mental health (lower CESD scores, lower GAD-7 scores and/or higher MHS scores). Illicit substance use was associated with higher CESD and GAD-7 scores and lower MHS scores.

For the psychotropic medication use outcomes, we first performed adjusted analyses in the absence of adjustment for CESD and GAD-7 scores. We found that marginal and low FS were associated with 2.06 (*p* < 0.001; 95% confidence interval [CI] = 1.36–3.13) and 1.99 (*p* < 0.01; 95% CI = 1.26–3.15) times higher odds of any psychotropic medication use, respectively, compared to high FS (Table 3). While very low FS was associated with 1.50 times higher odds of any psychotropic medication use, this was not statistically significant. Associations between FS and each individual category of psychotropic medication use exhibited a similar pattern of findings, with the adjusted odds ratios (AORs) consistently highest for marginal FS. Marginal FS was associated with 1.82 (*p* < 0.01; 95% CI = 1.19–2.80) times higher odds of antidepressant use and 1.73 (*p* < 0.05; 95% CI = 1.01–2.97) times higher odds of sedative use, while low FS was associated with 1.66 (*p* < 0.05; 95% CI = 1.03–2.65) times higher odds of antidepressant use. There were no significant associations between FS and antipsychotic use in these models, and no significant associations between very low FS and any of the psychotropic medication outcomes.

**Table 3.** Multivariable logistic regressions to examine the association between FS and psychotropic medication use among HIV+ women

	Any psychotropic medication use		Anti-depressant use		Sedative use		Anti-psychotic use	
	AOR	95% CI	AOR	95% CI	AOR	95% CI	AOR	95% CI
FS status (high ref)								
Marginal	2.06***	1.36–3.13	1.82**	1.19–2.80	1.73*	1.01–2.97	1.56	0.85–2.87
Low	1.99**	1.26–3.15	1.66*	1.03–2.65	1.72	0.96–3.10	1.21	0.60–2.45
Very low	1.50	0.88–2.53	1.18	0.68–2.04	1.35	0.68–2.69	0.95	0.43–2.11
Age at visit	1.04***	1.02–1.06	1.03**	1.01–1.05	1.04***	1.02–1.07	1.00	0.97–1.03
Race (non-Hispanic White ref)								
Hispanic	0.61	0.35–1.05	0.57	0.33–1.01	0.89	0.47–1.70	0.64	0.27–1.54
African-American/Black	0.35***	0.22–0.55	0.41***	0.26–0.65	0.40**	0.23–0.69	0.58	0.28–1.18
Other	0.79	0.36–1.74	1.21	0.55–2.66	0.29	0.08–1.04	0.61	0.15–2.39
Annual household income (⩽$12 000 ref)								
$12 001–24 000	0.52**	0.35–0.78	0.48***	0.31–0.74	0.80	0.48–1.33	0.68	0.38–1.22
⩾$24 001	0.57**	0.39–0.83	0.61*	0.41–0.91	0.48**	0.28–0.83	0.21***	0.10–0.47
Educational attainment								
High school degree or higher	1.01	0.73–1.42	0.90	0.64–1.27	0.99	0.64–1.53	0.77	0.47–1.26
Heavy drinking^a^								
Yes	1.18	0.73–1.90	1.20	0.74–1.95	1.04	0.54–2.00	0.68	0.30–1.54
Illicit substance use since last visit^b^								
Yes	1.05	0.61–1.83	1.25	0.72–2.17	0.83	0.40–1.72	1.93	0.92–4.02
Area under ROC curve	0.6858		0.6704		0.6824		0.6931	
Observations	867		867		867		867	

^∗∗∗^*p* < 0.001, ^∗∗^*p* < 0.01, ^∗^*p* < 0.05.

a Heavy drinking defined as ≥7 drinks/week (1 drink defined as 1 can, bottle, or glass of beer; 1 glass of wine; 1 shot of liquor on its own or in a mixed drink; or any other kind of alcoholic beverage).

b Participants were asked if they used cocaine, crack, heroin, methamphetamine, hallucinogens, club drugs, or any other illicit or recreational drugs not including cannabis since the last visit.

We next examined associations of FS with any psychotropic medication use, antidepressant use and sedative use, additionally adjusting for CESD and GAD-7 scores (Table 4). Marginal FS remained associated with 1.93 (*p* < 0.05; 95% CI = 1.16–3.19) times higher odds of any psychotropic medication use. The AORs for associations of marginal FS with antidepressant and sedative use were 1.64 and 1.42, respectively, although neither reached statistical significance. Associations between low FS and each psychotropic medication outcome were close to 1 (all not statistically significant), while very low FS was associated with lower odds of each outcome, with the association between very low FS and antidepressant use statistically significant (AOR = 0.42; *p* < 0.05; 95% CI = 0.19–0.96).

**Table 4.** Multivariable logistic regression models to examine the association between FS and psychotropic medication use among HIV+ women after adjusting for psychiatric symptoms

	Any psychotropic medication use		Anti-depressant use		Sedative use	
	AOR	95% CI	AOR	95% CI	AOR	95% CI
FS status (high ref)						
Marginal	1.93*	1.16–3.19	1.64	0.98–2.74	1.42	0.75–2.69
Low	1.07	0.61–1.89	0.99	0.55–1.77	0.95	0.46–1.96
Very low	0.52	0.24–1.12	0.42*	0.19–0.96	0.55	0.21–1.47
Age at visit	1.05***	1.03–1.07	1.04**	1.01–1.06	1.05**	1.02–1.08
Race (non-Hispanic White ref)						
Hispanic	0.84	0.44–1.63	0.78	0.40–1.53	1.25	0.56–2.82
African-American/Black	0.48**	0.28–0.82	0.52*	0.30–0.91	0.68	0.34–1.36
Other	0.67	0.25–1.81	1.03	0.38–2.78	0.17	0.02–1.40
Annual household income (⩽$12 000 ref)						
$12 001–24 000	0.45**	0.27–0.74	0.44**	0.26–0.74	0.68	0.37–1.25
⩾$24 001	0.73	0.46–1.16	0.81	0.50–1.30	0.58	0.30–1.11
Educational attainment						
High school degree or higher	1.16	0.77–1.76	1.00	0.66–1.54	1.02	0.60–1.72
Heavy drinking^a^						
Yes	0.93	0.52–1.67	0.97	0.53–1.78	1.20	0.57–2.53
Illicit substance use since last visit^b^						
Yes	0.74	0.37–1.47	0.83	0.41–1.68	0.65	0.27–1.57
CESD score	1.05***	1.02–1.07	1.04***	1.02–1.07	1.03*	1.00–1.06
GAD-7 score	1.05*	1.00–1.10	1.05*	1.00–1.10	1.07*	1.01–1.13
Area under the ROC curve	0.7358		0.7233		0.7322	
Observations	655		655		655	

^∗∗∗^*p* < 0.001, ^∗∗^*p* < 0.01, ^∗^*p* < 0.05.

a Heavy drinking defined as ≥7 drinks/week (1 drink defined as 1 can, bottle, or glass of beer; 1 glass of wine; 1 shot of liquor on its own or in a mixed drink; or any other kind of alcoholic beverage).

b Participants were asked if they used cocaine, crack, heroin, methamphetamine, hallucinogens, club drugs, or any other illicit or recreational drugs not including cannabis since the last visit.

Of the other variables, higher incomes were consistently associated with lower odds of psychotropic medication use prior to adjusting for CESD and GAD-7 scores. Having an annual income of $12 001–24 000 remained significantly associated with lower odds of any psychotropic medication use and antidepressant use after adjusting for CESD and GAD-7 scores, compared to having an annual income ⩽$12 000. Age was positively associated with any psychotropic medication use, antidepressant use and sedative use (but not antipsychotic use), both before and after adjusting for CESD and GAD-7 scores. Self-identifying as African-American/Black was associated with lower odds of any psychotropic medication use, antidepressant use and sedative use, compared to self-identifying as non-Hispanic White (although this was not significant for sedative use after adjusting for CESD and GAD-7 scores). In the fully adjusted model, CESD and GAD-7 scores were positively associated with any psychotropic medication use, antidepressant use and sedative use.

## Discussion

In this study of women with HIV in the USA, food insecurity was associated with the symptoms of common mental illness but displayed a complex relationship with psychotropic medication use. Similar to previous studies (Tuthill *et al.*, 2019; Whittle *et al.*, 2019b), we found a dose–response relationship between food insecurity and symptoms of common mental illness. We hypothesised that associations between food insecurity and psychotropic medication use would reflect this dose–response relationship, but our findings suggest a more complex picture. While marginal FS was associated with significantly higher odds of taking any psychotropic medication, antidepressants and sedatives, the magnitude of the associations decreased as severity of food insecurity increased. Very low FS was associated with lower odds of psychotropic medication use after adjusting for CESD and GAD-7 scores (although this only reached significance for antidepressant use).

While there may be several possible explanations for this pattern of findings, it is most likely that people who experience very low FS find it difficult to engage in mental health care because of competing resource demands. Such individuals may find it difficult to access mental health services and therefore have fewer chances to be prescribed psychotropic medications. Alternatively, they may access care but find it more difficult to adhere to medication regimens and subsequently have prescriptions discontinued. This possibility is supported by previous studies. Food insecurity has consistently been associated with poor engagement in care among PLHIV, including missing clinic visits and suboptimal adherence to medications (Young *et al*., 2014). In qualitative studies, food-insecure PLHIV describe how hunger, exhaustion, pre-occupation with finding food and less money for transport erect major barriers to attending clinics (Whittle *et al*., 2016). Similarly, in a nationally representative sample of non-elderly adults in the USA, individuals with severe mental illness who had very low FS were twice as likely to report being unable to afford mental health care, and 25% less likely to be using mental health services, compared to food-secure individuals with severe mental illness (Afulani *et al*., 2018).

Two studies in the USA have found that severe food insecurity was associated with higher rates of acute mental health care utilisation. Among outpatient users of mental health services, severe food insecurity was associated with five times the odds of having any psychiatric emergency room visit (Mangurian *et al*., 2013); and among a national sample of homeless adults, food insufficiency was associated with three times higher odds of psychiatric hospitalisation (Baggett *et al*., 2011). Poor access to ambulatory outpatient mental health services among severely food-insecure individuals may therefore result in inadequate long-term symptom control and, consequently, greater acute mental health care utilisation – which is the same pattern that has been seen in studies of food insecurity and HIV care (Weiser *et al*., 2013).

Another key finding is that marginal FS remained associated with nearly twice the odds of any psychotropic medication use after adjusting for symptoms of depression and anxiety. This supports our hypothesis that among these women living with HIV, food insecurity, at least in a milder form, may be associated with being prescribed psychotropic medications independent of symptoms of common mental illness. This suggests that people experiencing complex social problems such as food insecurity (but who do not experience such severe levels of social deprivation that they are unable to engage in care) may be prescribed psychotropic medications at a higher rate than those without such problems. This is supported by higher incomes also being associated with lower odds of psychotropic medication use in models additionally adjusted for CESD and GAD-7 scores. These findings indicate that there may be structural incentives and concomitant factors that favour the prescription of psychotropic medications for all forms of distress, regardless of the nature of the dominant contributing factors (e.g. biological *v*. psychological *v*. social). As explained above, these factors may include the clinical training of prescribers, the absence of resources for social interventions and/or the relative stability and provision that can accompany a psychiatric diagnosis through disability (Holmes, 2013; Hansen *et al*., 2014; Knight, 2015).

Given that the data were cross-sectional, these possible explanations must be interpreted cautiously, and further research is needed. It remains possible that the causality runs in the reverse direction: people with symptoms of common mental illness significant enough to warrant treatment with psychotropic medications may be more likely to be food-insecure, and then may be referred to food assistance services that prevents them from experiencing the most severe form of food insecurity. Longitudinal studies investigating directionality and dominant mediating mechanisms are needed to comprehensively understand this association.

Our study has other limitations. It is not clear from the data who was prescribing psychotropic medications for these women (e.g. HIV specialists *v*. psychiatrists). Greater clarity on this aspect of the findings would be helpful. Similarly, we have no data on access to mental health services and attendance at clinic appointments, which would clarify some of the mechanisms and explanations behind the findings. Third, we have no data on other mental health treatment modalities among these women, and no data on adherence to treatments, whether pharmacological or non-pharmacological. Measurement of these variables will be important for future studies. Finally, we were unable to adjust for any other symptoms of mental illness besides depression and anxiety because such data were not collected in the WIHS.

## Conclusion

Our findings reveal a complex relationship between food insecurity and psychotropic medication use among women living with HIV in the USA. The data tentatively suggest that (1) PLHIV with severe food insecurity may face barriers to engaging in mental health services, and (2) food-insecure PLHIV who are able to engage in mental health services may be more likely to be prescribed psychotropic medications. While these interpretations come with significant caveats owing to limitations of the dataset, they warrant further research, including longitudinal and qualitative studies, examining the relationships of food insecurity (and other forms of social deprivation) with symptoms of common mental illness, access to mental health services, and the prescription of psychotropic medications and other treatment modalities. If further substantiated, our findings support recent calls for incorporating more detailed understandings of the social-structural determinants of health into clinical curricula (Metzl and Hansen, 2014; Hansen *et al*., 2018) and for re-examining how the structure of the US social safety net and healthcare system affects the course of different chronic illnesses (Hansen *et al*., 2014; Whittle *et al*., 2017).

## Data

Data for this manuscript were provided by the Women's Interagency HIV Study (WIHS) as part of an Executive Committee (EC) approved concept sheet. These data cannot be provided to other investigators or used for additional projects without the written consent of the WIHS EC. Data are made available to investigators through a formal concept sheet submission and review process or the WIHS Public Use Data Set. Please visit http://www.wihshealth.org for more information on how to obtain WIHS data.
